# The Conformational Switches of a Bacterial Light‐Driven Sodium Pump Characterized by Time‐Resolved Resonance Raman Spectroscopy

**DOI:** 10.1002/cphc.202500905

**Published:** 2026-04-09

**Authors:** Anna Lena Schäfer, Arita Silapetere, Peter Hegemann, Katrina T. Forest, Uwe Kuhlmann, Peter Hildebrandt

**Affiliations:** ^1^ Institut für Chemie Technische Universität Berlin Berlin Germany; ^2^ Institut für Biologie Humboldt‐Universität zu Berlin Berlin Germany; ^3^ Department of Bacteriology University of Wisconsin‐Madison Madison Wisconsin USA

**Keywords:** photocycle, resonance Raman, retinal protein, sodium pump, time‐resolved

## Abstract

*Krokinobacter eikastus* rhodopsin 2 (KR2) is a microbial light‐driven ion pump that transports Na^+^ out of the cell. The active transport is coupled to a photocycle of the retinal chromophore, covalently attached to the protein via a Schiff base linkage. In this work, we have employed time‐resolved pump‐probe resonance Raman (TR RR) spectroscopy to characterize the operational switches of KR2 that control Na^+^ uptake from the cytosol and release to the exterior. The analysis was based on a comprehensive spectroscopic investigation of the parent state using different excitation lines. Two substates were identified in which the Asp116 counterion and water positions differ with respect to the Schiff base. In line with published crystallographic data, one of the substates was ascribed to the active configuration that binds Na^+^ after deprotonation of the Schiff base. The main advantage of the present TR RR method is that chromophore structures of the photocycle intermediates and their kinetics can be determined. Thus, we identified two redshifted intermediates O1 and O2 formed within ca. 2 and 5–7 ms after illumination, in which the chromophore adopts a protonated 13‐*cis* and all‐*trans* configuration, respectively. This isomerization is ascribed to be the switch for Na^+^ release.

## Graphical abstract

The microbial retinal protein KR2 *(Krokinobacter eikastus)* is a light‐driven outward Na^+^ pump, which is coupled to the photocycle of the chromophore. Using time‐resolved continuous‐wave pump‐probe resonance Raman spectroscopy, the operational switches for ion transport are identified, which are the transitions to a specific Schiff base sub‐state and the precursor of the dark state for Na^+^ uptake and release, respectively.

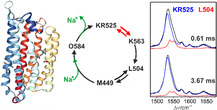

## 1 Introduction

Microbial rhodopsins constitute a family of membrane proteins that include a retinal chromophore covalently attached to the protein via a Schiff base linkage [1]. These photoreceptors utilize light either for energy conversion or signaling. Energy converters function as ion pumps to generate an ion gradient across the membrane. Ion pumping is driven by a photoinduced cyclic process of the retinal chromophore. The classical example is bacteriorhodopsin (BR), an archaeal proton pump discovered more than 50 years ago [2]. Meanwhile, also anion and cation pumps have been found in archaea and bacteria. Among them is the *Krokinobacter eikastus* rhodopsin 2 (KR2), which functions as a light‐driven outward sodium ion pump in its natural marine Na^+^‐rich habitat [3, 4, 5]. Many mechanistic and structural properties of KR2 are similar to BR. Moreover, the knowledge about the molecular functioning of BR that was accumulated over several decades substantially supported and guided the research on the recently discovered KR2.

**FIGURE 1:**
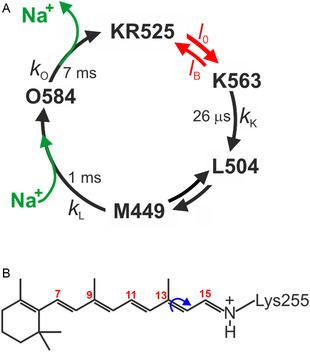
Photocycle and retinal chromophore in KR2. (A) Simplified scheme of the photocycle of KR2. Spectral and kinetic data was taken from the literature [4, 6, 7, 8]. The approximate decay times refer to pH 8.0 and 100 mM Na^+^ for detergent‐solubilized KR2. The individual states are indicated by commonly used notation with the numbers indicating the absorption maxima. The photochemical and thermal reactions are characterized by red and black arrows/symbols, respectively. The green arrows indicate uptake and release of Na^+^. (B) The retinylidene chromophore in the all‐*trans*, 15‐*anti* configuration in the dark state KR525. The protonated Schiff base is formed by attachment to Lys255. The blue arrow indicates the isomerization site.

Figure 1 shows the photocycle of KR2 as determined by absorption spectroscopy [4, 6, 7, 9].

The dynamics of these processes were studied in detail by ultrafast spectroscopy and flash photolysis [8, 10, 11, 12]. Following light absorption in the dark state KR525, the all*‐trans* retinal chromophore isomerizes around the C(13)=C(14) bond to yield a distorted 13*‐cis* configuration in the K563 state [4]. Subsequent relaxation of the chromophore and its immediate surrounding leads to the formation of L504, which forms a rapid equilibrium with M449 in which the Schiff base is deprotonated. The decay of M449 to O584 is associated with the reprotonation of the 13‐*cis* retinal Schiff base and binding of Na^+^ from the cytoplasmic side [4]. Na^+^ release to the extracellular side takes place with the decay of O584.

In electrical experiments, the kinetics of the photocycle correlated with the internal cellular Na_i_^+^ concentration and the recovery time of KR525 varies between 7 ms at 110 mM Na_i_^+^ and 17 ms at 0.1 mM Na_i_^+^ [13].

As revealed by comparison with other members of this retinal protein family, Na^+^ pumping is associated with the conserved Asn112, Asp116, and Gln123 residues (NDQ triad) on the third transmembrane helix (h3), which corresponds to the DTD motif in archaeal outward proton pumps [3, 14, 15]. Additional residues like His30, Ser70, Arg109, Asp251, Ser254, and Gly263 were identified as critical for sodium pumping [3, 16, 17].

The elucidation of the molecular mechanism of KR2 requires structural models, which have been obtained by cryo‐crystallography and cryo‐electron‐microscopy [16, 18, 19, 20, 21, 22]. Furthermore, time‐resolved serial femtosecond crystallography provided insight into the structural dynamics of the KR2 photocycle [23, 24]. These structures constitute an indispensable reference for the interpretation of spectroscopic results.

According to the current understanding, one region of the protein undergoes functionally critical changes of the structure: the Schiff base of the chromophore and its immediate environment. Here, NMR [25, 26, 27], IR [28, 29, 30, 31, 32], and resonance Raman (RR) spectroscopic techniques [10, 15, 33, 34, 35, 36, 37, 38] were employed to complement and refine the crystallographic data. Among them, the seminal RR spectroscopic studies by the groups of Kandori and Unno represent substantial contributions to understanding the molecular functioning of sodium pumps (vide infra) [15, 33, 34, 35, 36, 38]. Furthermore, they identified key issues to be addressed in future studies, particularly the role of hydrogen bonding and electrostatic interactions of the Schiff base [38], and the molecular events associated with formation and decay of O584 [36].

In this work, we have addressed these issues using time‐resolved (TR) RR spectroscopy. We have recently adapted the classical continuous‐wave (cw) pump‐probe arrangement [39, 40] to a confocal Raman spectroscopic setup [41]. This approach offers the advantage that the essential boundary conditions for TR RR experiments can be clearly defined, i.e., the fresh sample conditions and photochemical innocence of the probe beam, as well as the delay time between pump and probe events. On the basis of this approach, we identified two structurally different red‐shift intermediates that are presumably associated with the switch to release Na^+^. Furthermore, we elucidated the nature of the two different “Schiff base–counterion” configurations [18], which may serve as a conformational switch for Na^+^ uptake.

## 2 Experimental

### 2.1 Protein Handling

KR2 was overexpressed in *Escherichia coli* and purified as described in the Supporting Information (Section S1; Figure S1), following previously published procedures [13, 18]. For spectroscopic measurements, the protein (100–150 μM) was solubilized in detergent (0.02% DDM and 0.004% CHS) at pH 8.0 (50 mM Tris‐HCl, 150 mM NaCl, 5% Glycerol, 0.1 mM PMSF). For a comparative experiment, the protein was incorporated in phospholipid vesicles at the same pH and salt concentration (Section S1).

### 2.2 RR Spectroscopy

RR spectra were recorded using a confocal Raman spectrometer (Horiba, LabRam HR‐800) equipped with a liquid‐nitrogen cooled CCD detector (Horiba, LN‐BIUV‐2048). The spectral resolution was ca. 2 cm^−1^, and the wavenumber increment per pixel was ca. 0.5–0.7 cm^−1^, depending on the excitation line. The probe laser line was focused onto the sample to a spot of 4 μm diameter using a microscope objective with a numerical aperture of 0.35. All measurements were conducted at ambient temperature using a rotating cell including the sample solution. Kr and Ar ion cw lasers (Coherent, CR and Innova series) with wavelengths at 458, 488, 514, 568, and 647 nm were used for pumping and probing. The pump‐probe set was described in detail previously, including a comprehensive discussion of the conditions to fulfill the key criteria for TR RR experiments, i.e., the fresh sample condition and the photochemical innocence of the probe beam. Accordingly, we have adapted the experimental parameters (e.g., laser power, rotational frequency of the cell) as described in the Supporting Information (Section S2). Spectra were calibrated to an accuracy of ca. 0.5 cm^−1^ based on the Raman bands of toluene as a reference. Data processing, such as baseline corrections, was conducted using the OPUS 7.8 (Bruker) software and in‐house programs. No smoothing procedures were applied to the experimental spectra. Band fitting using Lorentzian band shapes and component analysis was carried out using in‐house programs [42].

## 3 Results

### 3.1 RR Spectroscopy of the Dark State

The RR spectrum of the parent state of KR2 was measured in probe only‐experiments with 458, 488, or 568 nm excitation. These spectra were obtained in the absence of an additional pump beam. Setting the rotational frequency of the cuvette ν_0_ to 20 s^−^^1^, the fresh sample condition $\frac{k_{\mathrm{RL}}}{\nu_{0}}\geq 4$ is fulfilled [41], taking into account the recovery time of KR2 of ca. 7 ms as the rate‐limiting step of the photocycle *k*_RL_ (=*k*_O_) reported for the present experimental conditions (Figure 1). However, the TR RR experiments in this work point to a slightly longer recovery time (vide infra), albeit still close to satisfying the fresh sample condition with ν_0_ = 20 s^−^^1^. Using a laser power P_0_ of 0.1 mW for 458 and 488 nm excitation, the extent of photoconversion by the probe beam is, on average, 2% and 5%, respectively (see Section S2). Any spectral contribution of K563 and L504 in the probe beam can safely be neglected due to the weak resonance enhancement and slow rate of formation, respectively. For 568 nm excitation we have used a power of 1 mW, corresponding to a slightly higher conversion to K563 (8%) which, due to the good match of excitation line and electronic transition, might cause a small spectral contamination of the RR spectrum.

### 3.2 Chromophore Structure of the Parent State

**FIGURE 2:**
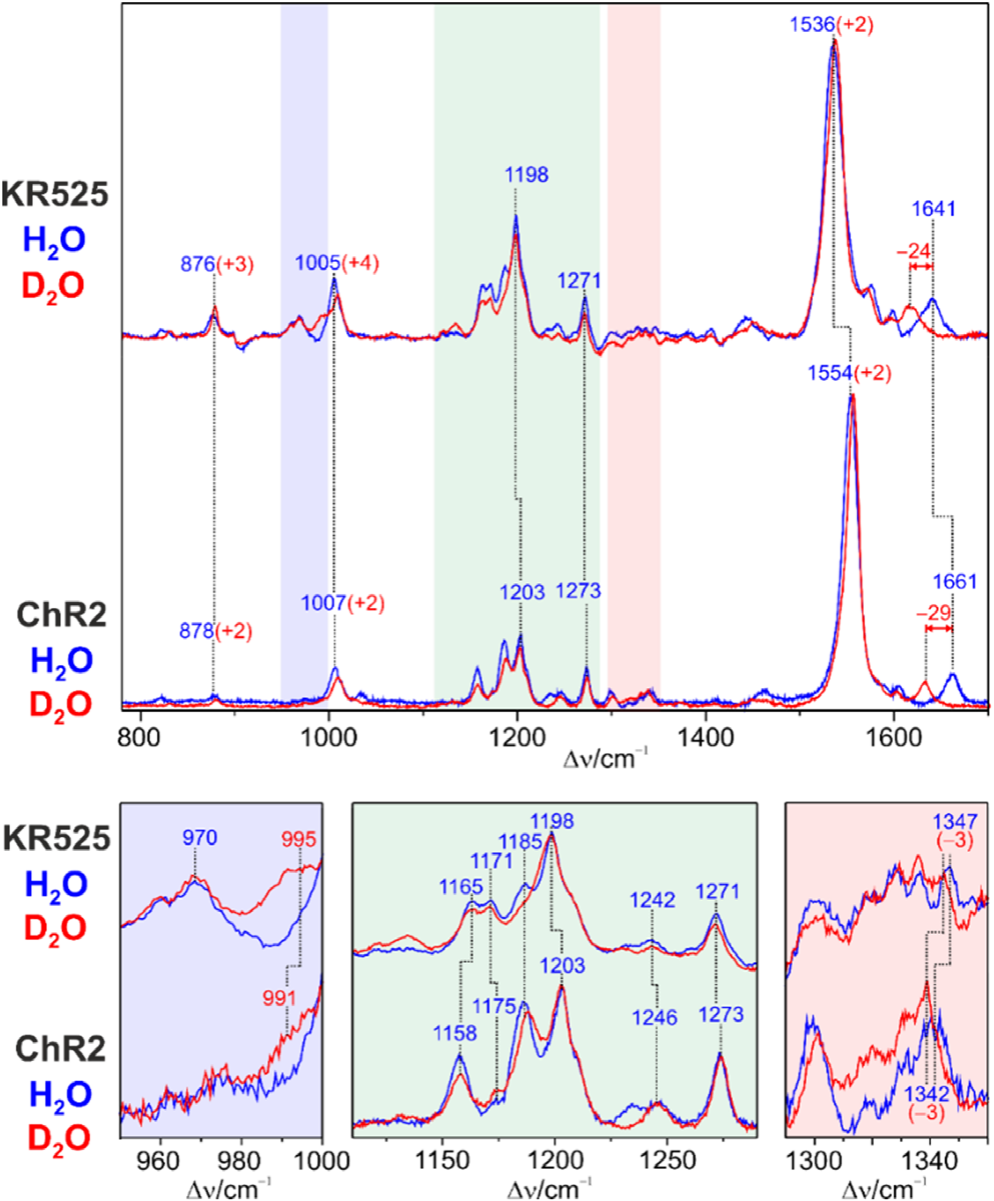
RR spectra of the dark state of KR2 and the all‐*trans*, 15‐*anti* configuration of the H134R variant of ChR2, measured in H_2_O (blue) and D_2_O (red) using 458 nm excitation. The light blue‐, green‐, and red‐highlighted regions in the top spectra are shown in close‐up views at the bottom. The spectra of ChR2 were reported in a previous work [43]. The red numbers in parentheses indicate the frequency shifts in D_2_O with respect to the bands in H_2_O labeled by the blue numbers.

In Figure 2, the RR spectra of KR525 measured in H_2_O and D_2_O are compared with those of the all‐*trans*, 15‐*anti* retinal Schiff base configuration of the H134R variant of channelrhodopsin‐2 (ChR2). This configuration and the 13‐*cis*, 15‐*syn* configuration form a photostationary equilibrium at low light intensities (denoted as apparent dark‐adapted state) [43].

The vibrational band pattern agrees very well with that of KR525. The region between 1150 and 1300 cm^−^^1^, considered as the fingerprint for the retinal conformation and configuration [44, 45, 46, 47], shows far‐reaching similarities with only minor deviations of the band positions. This confirms the view of an all‐*trans*, 15‐*anti* configuration of the protonated retinylidene Schiff base [25], as it was also demonstrated for the dark state of the proton pump BR or anion‐pump halorhodopsin [45, 48, 49, 50, 51]. Unlike the apparent dark‐adapted state of ChR2 in which the all‐*trans*, 15‐*anti* and 13‐*cis*, 15‐*syn* configurations coexist at a ratio of 3:1 [43], in KR525 a contribution of the 13‐*cis*, 15‐*syn* configuration must be very small as indicated by the weak band at 1185 cm^−^^1^ [46]. A notable 13‐*cis*, 15‐*syn* contribution of more than 10% can be ruled as also shown by the spectral effects of H/D exchange. In contrast to the 13‐*cis*, 15‐*syn* configuration [46], the N—H in‐plane bending (ip) coordinate is only poorly coupled with C—H ip and C—C stretching coordinates in the all‐*trans* configuration as indicated by the lack of H/D shifts in the fingerprint region. Instead, coupling is largely restricted to two modes. One mode gives rise to the very weak band at 1347 cm^−^^1^, and involves the C(15)—H ip and N—H ip coordinates [44, 45, 50]. This coupling is removed upon H/D exchange leading to largely pure C(15)—H ip and N—D ip modes at 1344 and 995 cm^−^^1^, respectively. The second mode that includes the N—H ip coordinate is the C=N stretching at 1641 cm^−^^1^. Upon H/D exchange and thus removal of the coupling, the C=N stretching shifts down to 1617 cm^−^^1^. Due to the coupling with the N—H ip, both the frequency of the C=N stretching and the magnitude of the H/D downshift increase with increasing hydrogen bond strength of the protonated Schiff base [52].

Unlike the dark states of ChR2 and BR, KR525 displays a striking RR activity of the hydrogen‐out‐of‐plane (HOOP) mode at 876 cm^−^^1^, which is slightly sensitive to H/D exchange (Figure 2). This supports the assignment to the C(14) HOOP mode with minor contributions from the adjacent N—H HOOP coordinate [45]. In a planar chromophore structure, the HOOP modes are Raman‐inactive but this selection rule does not hold for distorted geometries, pointing to a twisted C(15)=N bond as proposed previously [33].

**FIGURE 3:**
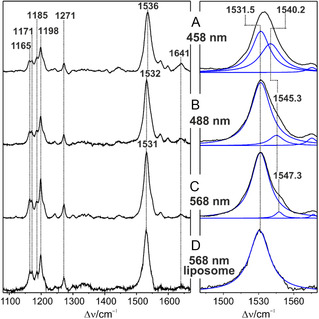
RR spectra of the dark state of KR2 measured at different excitation wavelengths in H_2_O (pH 8.0). (A) 458 nm, 0.1 mW; (B) 488 nm, 0.1 mW; (C,D) 568 nm, 1.0 mW. The spectra (A–C) were measured from the detergent‐solubilized protein, whereas spectrum (D) was obtained from KR2 incorporated into phospholipid vesicles. In spectrum (D), artifacts, presumably due to stray light, were removed by hand. The original spectrum is shown in Figure S2.

The RR spectra of the dark state of KR2 were measured with different excitation lines at both sides of the first allowed electronic transition (Figure 3). The relative RR band intensities vary only slightly, indicating the same resonance enhancement mechanism, corresponding to a Franck–Condon mechanism as described by Albrecht's A‐term [53]. Furthermore, the spectra of KR2 solubilized in detergents and incorporated in phospholipid vesicles agree very well and thus rule out that protein–lipid interactions influence the chromophore structure.

A striking observation, however, refers to the prominent peak in the C=C stretching region since it reveals small but clearly detectable frequency shifts from 1536 cm^−^^1^ at 458 nm to 1532 cm^−^^1^ at 488 nm and 1531 cm^−^^1^ at 568 nm excitation, concomitant to changes of the bandshape. The spectrum of KR525 in liposomes at 568 nm excitation represents a limiting case since the bandshape of the C=C stretching is highly symmetric. For the other three spectra, band fitting requires two components to achieve a satisfactory simulation of the experimental spectra. The low‐frequency component was found to be at 1531.5 ± 0.5 cm^−^^1^ in all four spectra, whereas the frequency of the high‐frequency component steadily increases from 1540.2 to 1547.3 cm^−^^1^ with increasing excitation wavelength. In particular, the fits to the spectra at 458 and 488 nm do not lead to unique minima. This is in line with the fact that also the second derivatives do not provide a hint for the approximate position of the high frequency component (Figure S3). Nevertheless, the analysis unambiguously demonstrates two closely spaced C=C stretching modes.

On the one hand, one may attribute these bands to two different normal modes that include the C=C and C—C stretching coordinates of the retinal chain [44, 45]. In all‐*trans* isomers, these two modes are predicted to have very similar frequencies but typically only one of them is RR active. Conversely, in 13‐*cis* isomers both modes are observed in the RR spectra [51]. It might be that in the present case, the originally Raman‐inactive mode gains intensity due to the distortion of the chromophore geometry in the vicinity of the Schiff base. A similar effect has been observed for β‐carotene, where the two closely spaced modes could be identified on the basis of slightly different excitation profiles, attributed to Dushinsky rotation [54].

**FIGURE 4:**
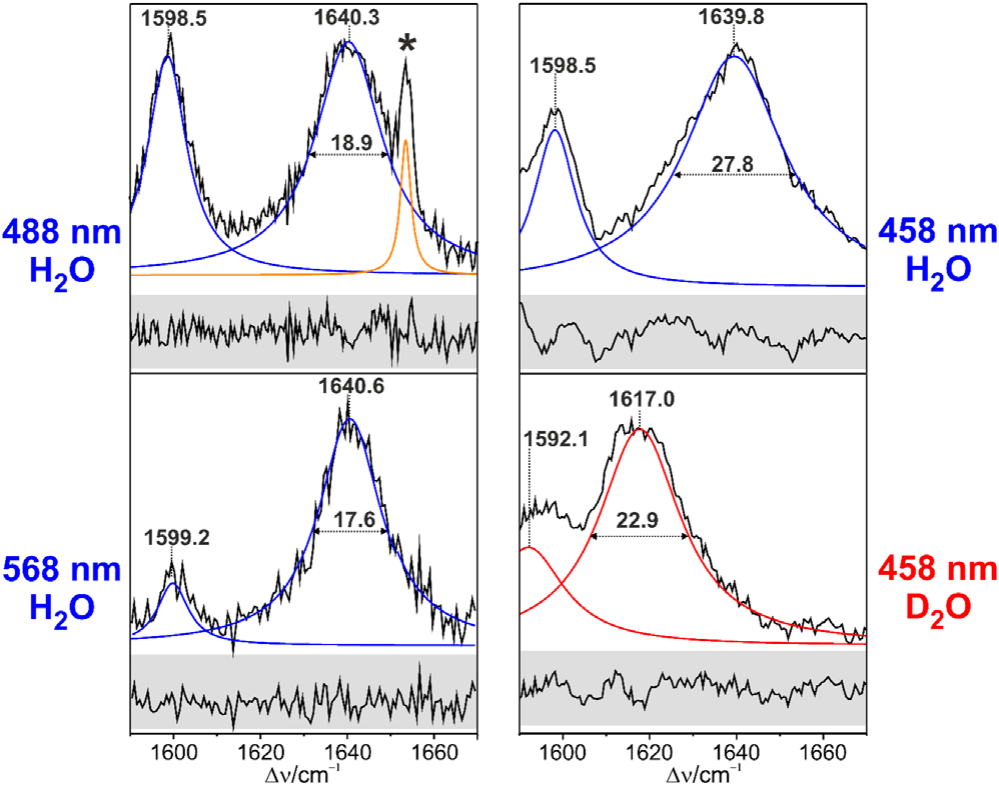
RR spectra of the dark state of KR2 measured at different excitation wavelengths in H_2_O (pH 8.0) and D_2_O (pD 8.0). Frequencies and half widths of the fitted Lorentzian functions are indicated. The spectrum measured with 488 nm excitation includes a nonlasing emission line of the Ar^+^ ion laser marked by an asterisk. The gray‐shaded boxes include the residuals of the fits.

However, this explanation cannot account for the substantial differences in the Raman excitation profiles of KR525 since the high frequency component nearly vanishes at 568 nm excitation but steadily increases upon shifting the excitation wavelength to higher energy. Therefore, as an alternative explanation, each of the two band components may be attributed to a specific all‐*trans*, 15‐*anti* substate. These two substates of KR525 exhibit essentially the same chromophore structure but differ with respect to electrostatic interactions, most likely via the Schiff base and its counterion Asp116 [3]. Support for this interpretation is derived by the analysis of the C=N stretching mode (Figure 4).

The fit of Lorentzian bandshapes reveals that the C=N stretching frequency slightly decreases and the half width increases upon shifting the excitation line from 568 nm to shorter wavelengths. These effects are clearly larger than the intrinsic experimental and fitting error. In particular, the increase of the half width from 488 nm (18.9 cm^−^^1^) to 458 nm (27.8 cm^−^^1^) is striking. Furthermore, for 458 nm excitation, the half width of the C=N stretching strongly decreases upon H/D exchange, similar to previous observations for BR and ChR2 [43, 55].

### 3.3 Pump‐Probe Experiments

**FIGURE 5:**
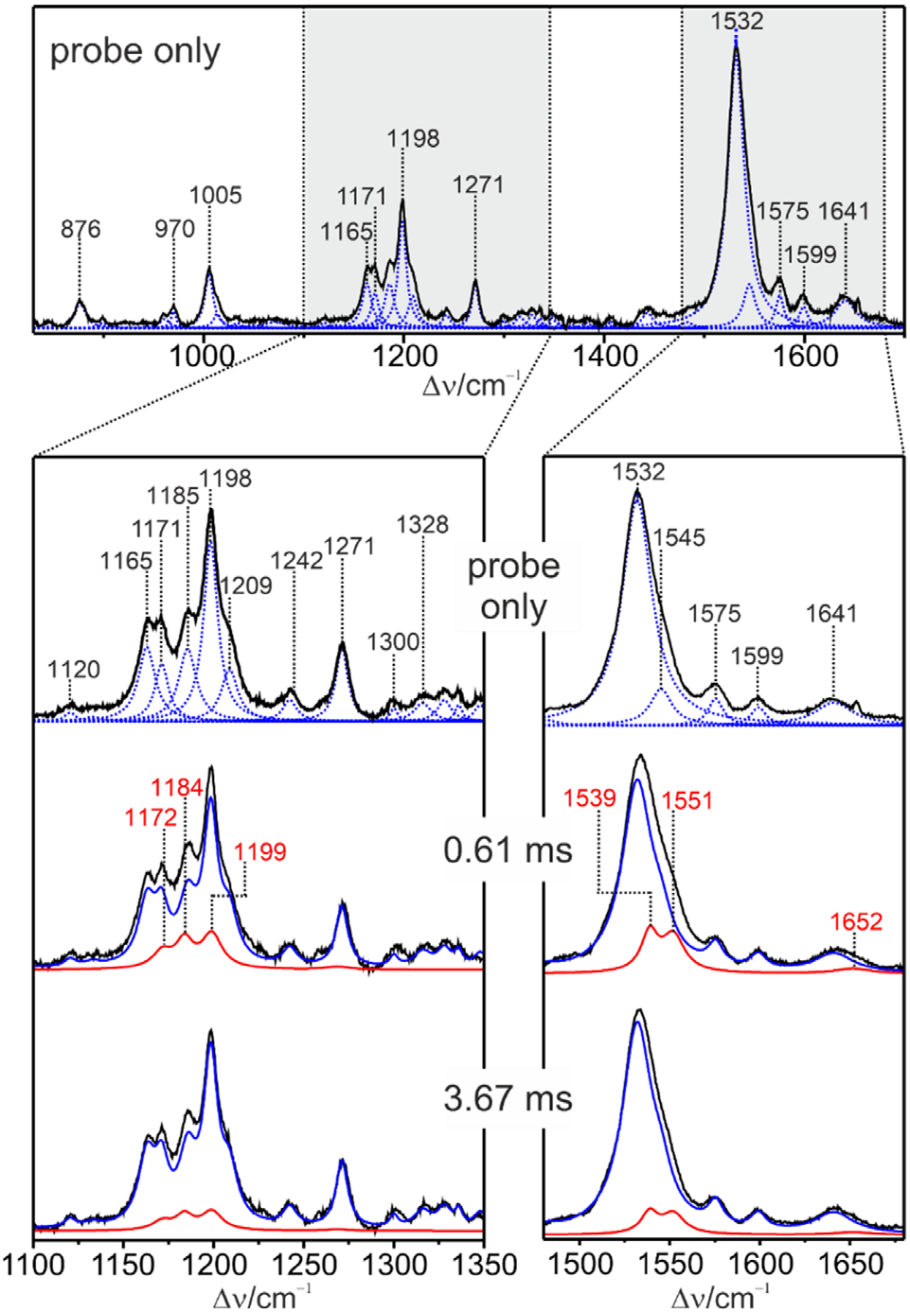
TR RR spectra of KR2 with 488 nm excitation (0.1 mW) of the parent state (probe only—top) and with the additional pump beam (568 nm, 100 mW) at different delay times. The gray‐shaded rectangles in the top spectrum correspond to the fingerprint and C=C stretching region, which are shown below in an expanded view. The parent state spectrum was analyzed by a band fitting using Lorentzian lineshapes (dotted curves) which were then used to generate the component spectrum of KR525 (blue solid line). The TR RR spectra, including the component spectra of KR525 and L504 (red solid line), are shown for two delay times. Further spectral data are reported in the Supporting Information, Figure S4.

To initiate the photocycle, we used the 568 nm line for excitation with maximum output leading to a power of 100 mW at the sample. Spectra were measured at room temperature for 16 delay times with a probe wavelength of 514 nm. A selection of the spectra is shown in Figure 5. The spectra were subjected to a component analysis [42], which started with the generation of the component spectrum of the parent state from the probe‐only spectrum. Here, Lorentzian functions were fitted to the spectrum and the resultant set of bands defined the component spectrum of KR525. This component spectrum and a set of additional bands were then used to simulate all TR pump‐probe RR spectra. In a global fit, the spectral parameters of the additional bands were refined to generate component spectra of the intermediate. In fact, only one intermediate, i.e., L504, was identified in this way, whereas any attempts failed to detect also M449 even on the basis of its strongest RR band expected at a frequency between 1560 and 1570 cm^−^^1^ [43, 51, 56, 57]. Most likely, the relative concentration of M449 was too low for a RR spectroscopic observation [3, 7, 15], particularly since, in general, the extent of photoconversion by the pump beam was relatively small in the confocal setup (Figure 5) [41]. The primary photoproduct K563 was not identified in the spectra due to its short lifetime and the poor resonance conditions (Figure 1).

**FIGURE 6:**
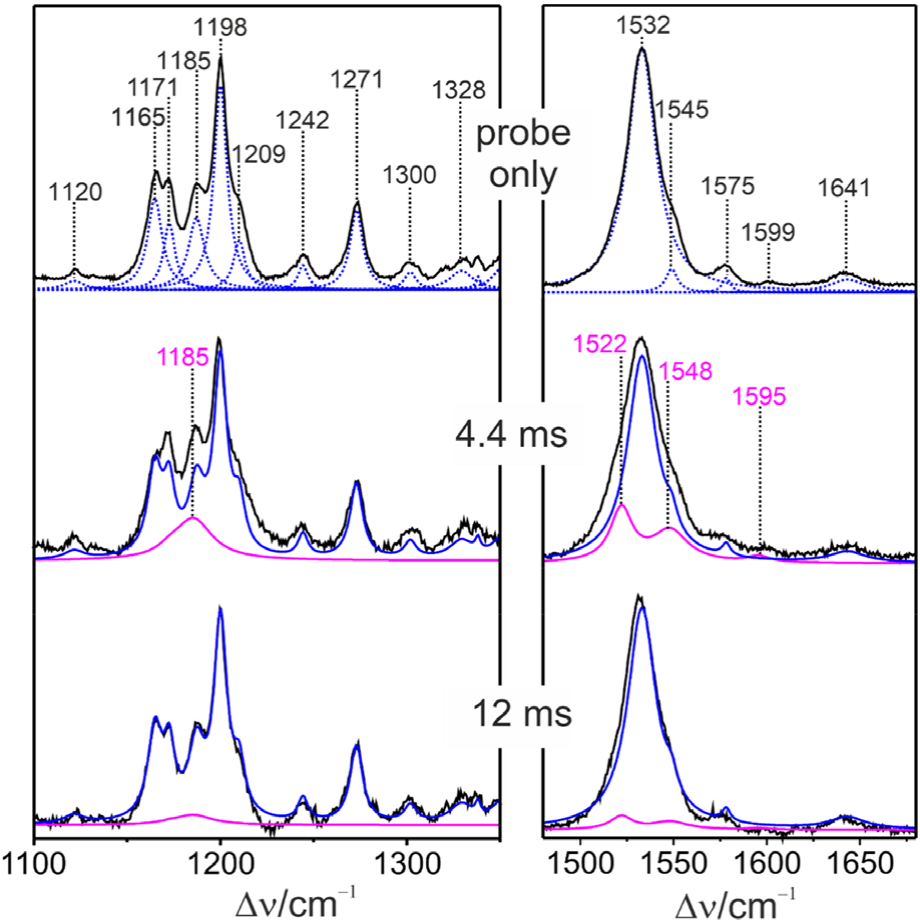
TR RR spectra of KR2 with 568 nm excitation (1.0 mW) of the parent state (probe only) and with the additional pump beam (514 nm) at different delay times. The average content of intermediates in the probe beam is estimated to less than 8% despite the laser power of 1 mW that was higher than with 488 nm excitation (Section S2). The parent state spectrum was analyzed as described above (Figure 5). The component spectra of KR525 and O584 are displayed in blue and magenta solid lines. Further spectral data are shown in Figure S5.

The second series of measurements was directed to monitor the temporal evolution of the O584 intermediate. Among the possible pump‐probe wavelength combinations that were tested, a probe wavelength of 568 nm (1 mW) and a pump wavelength of 514.5 nm (500 mW) turned out to be most efficient. The rotational frequency was kept unchanged (20 s^−1^). Also, these spectra were evaluated by the component analysis as described above (Figure 6).

### 3.4 Kinetic Analysis

**FIGURE 7:**
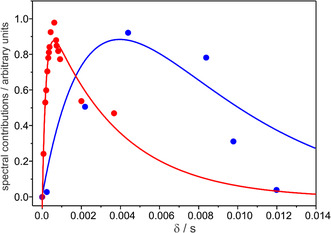
Kinetic analysis of the relative spectral contributions of L504 (red circles) and O584 (blue circles) derived from two series of TR RR spectroscopic measurements. The data for L504 and O584 were scaled to the same maximum values. The data sets for L504 and O584 were simulated separately by a two‐exponential function according to the kinetic schemes $K563\;\overset{k_{K}}{\to }\;L504\;\overset{k_{L}}{\to }\;O584$ and $L504\;\overset{k_{L}}{\to }\;O584\;\overset{k_{O}}{\to }\;\mathrm{KR}525$, respectively. The rate constants are listed in Table 1.

From the TR RR spectra measured at the delay times of between 20 μs and 12 ms, we thus determined the relative spectral contributions, which were used to evaluate the kinetics (Figure 7). The relative spectral contributions should not be confused with relative concentrations. In a crude approximation, one may estimate that the maximum amount of photoconverted KR2 does not exceed 15% of the total protein concentration. Since the spectral contributions for L504 and O584 were obtained from different series of measurements, we multiplied the data of L504 by a factor such that the maxima of the fitted functions of L504 and O584 were matched. The rate constants determined by the fits are listed in Table 1. The average error of the rate constants is ±17%. This relatively large error is related to the small spectral contributions of the intermediates [41]. For instance, the maximum spectral contribution of L504, observed at a delay time of 0.61 ms, is still ca. 4 times smaller than that of the parent state.

**TABLE 1:** Rate constants and decay times of the photocycle of KR2 derived from TR RR spectroscopy.

Reaction	Rate constant/decay constant	This work^a^		Refs. [3, 6, 8]
		L504 data	O584 data	
K563 → L504	*k*_K_/s^−^^1^	5.3⋅10^3^		4.0⋅10^4^–5.6⋅10^4^
	*τ*_K_/ms	0.19		0.025–0.018
L504 → O584^b^	*k*_L_/s^−^^1^	2.7⋅10^2^	3.3⋅10^2^	1.0⋅10^3^–6.0⋅10^3^
	*τ*_L_/ms	3.7	3.0	1–0.17
O584 → KR525^c^	*k*_O_/s^−^^1^		1.9⋅10^2^	1.3⋅10^2^–1.5⋅10^2^
	*τ*_O_/ms		5.3	7.7–6.7

a The rate constants were determined separately for the individual datasets, based on a simplified reaction scheme (Figures 1 and 7). The average error was ±17%.

b Corresponds to the reaction of L504/M449 to O1.

c Corresponds to the reaction of O2 to KR525.

The datasets derived from the evolution of L504 and O584 were analyzed separately, using a two‐exponential function in each case (Figure 7). Thus, the fits yielded two values for *k*_L_, which agree within the experimental and fitting accuracy. However, for the formation and decay of L504, the rate constants are smaller than the reported data derived from transient absorption spectroscopy by a factor of ca. 10 [3, 6, 8].

These deviations cannot exclusively be explained by an overestimation of L504 when its contribution is small, i.e., at early and late times of its formation and decay, respectively. In addition, they reflect deficiencies of the underlying kinetic models, specifically the neglect of M449 in the present analysis (Figure 1) [4, 8]. This intermediate, which according to flash photolysis data forms a rapid equilibrium with L504, was not identified in the TR RR experiments and, hence, not considered in the present kinetic model. Furthermore, the analysis is based on one O584 component spectrum, which—as will be shown below—is a simplification albeit unavoidable in view of the limited number of experimental data.

### 3.5 Chromophore Structure in the Intermediate States

**FIGURE 8:**
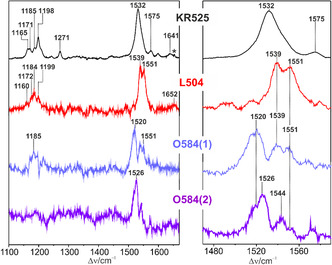
Spectra of KR525 and the intermediate states. The left and right panels shown an overview of the spectra and a close‐up view of the C=C stretching region, respectively. The spectrum of KR525 was measured as described in Figure 5 (488 nm). The symbol * denotes a nonlasing plasma line of the Ar^+^ ion laser. The spectra of L504 were generated by subtracting the parent state contribution from the sum of several TR RR spectra using the 488/568‐nm probe/pump combination as described in Figure 5. In the same way, we extracted the spectrum of O584 from the TR RR spectra with 568‐nm probe and 514‐nm pump beams (Figure 6). In this case, however, the O584 spectra at 4.4 and 12 ms were different. These spectra, denoted as O584(1) (4.4 ms) and O584(2) (12 ms), do not correspond to pure states.

We now compare the spectra of the KR2 parent state and the intermediates (Figure 8). The RR spectrum of L504 was generated from the sum of various TR RR spectra after subtraction of the parent state contribution. Whereas the “early” and “late” L504 intermediate reveals nearly identical RR spectra (Figure S6), there are notable differences between the O584 spectra derived from the TR RR measurements at delay times of 4.4 (early) and 12 ms (late) (Figures 8 and S7). Both spectra at 4.4 and 12 ms, denoted as O584(1) and O584(2), respectively, will be discussed in the following.

Following photoisomerization of the C(13)=C(14) bond of the all‐*trans* retinylidene Schiff base in KR2, the photoproduct K563 relaxes to L504. The L504 chromophore thus exhibits a 13‐*cis* configuration and its RR spectrum is very similar to the L550 intermediates of BR or halorhodopsin [47, 48, 51, 58, 59]. All these species exhibit the characteristic double‐banded structure in the C=C stretching region, with components at 1539 and 1551 cm^−^^1^ in L504, and the upshifted C=N stretching mode from 1641 (KR525) to 1652 cm^−^^1^ (L504). These similarities argue against a simultaneous isomerization of the C(13)=C(14) bond and the Schiff base in the primary photoprocess, which in addition has never been observed for ion pump and channel retinal proteins.

L504 forms an equilibrium with M449 [7], which we were unable to detect, presumably due to its low relative concentration (vide supra). The couple L504/M449 then decays to O584 [7]. The two RR spectra of O584, measured at *δ* = 4.4 ms (O584(1)) and 12 ms (O584(2)), differ from each other but do not reflect pure states. In O584(1), the two bands at 1539 and 1551 cm^−1^ agree very well with the strongest bands of L504, which in fact may still comprise ca. 40% of the maximum intermediate concentration at 4.4 ms according to the kinetic data in Figure 7. Thus, the 1539/1551 cm^−1^ doublet is attributed to L504. Accordingly, the main RR band that can unambiguously be attributed to an O584 state is the relative broad 1520 cm^−1^ band which is due to the C=C stretching (Figure 8). Its low frequency is in line with an absorption maximum at 584 nm [60]. The assignment of the broad 1185 cm^−1^ band envelope is not straightforward. Its position is similar to the corresponding band in L504, but in view of its relative intensity similar to the 1539 and 1551 cm^−1^ bands, it cannot completely be due to L504 but must partly also originate from O584. In the RR spectrum of O584(2), the bands of L504 have disappeared and also the intensity of the 1520 cm^−^^1^ band is decreased. Instead, a new band at 1526 cm^−1^ band is observed. Hence, we conclude that L504 is followed by the early O584 species with bands at 1520 and 1185 cm^−^^1^ which is then converted to the late O584 (1526 cm^−1^) as the precursor for KR525. A more comprehensive discussion of the intermediates following L504 is not possible in view of the low signal‐to‐noise ratio. For this reason and due to the low relative spectral contributions of the intermediates, we also did not analyze the 568 nm probe/514 nm pump TR RR spectra (Figure 6) by explicitly considering two O584 and the L504 component spectra. Instead, these intermediates were represented by one component spectrum (Figure S7). Hence, the resultant error may be comparable to those of previous flash‐photolysis studies which as well did not discriminate between different O584 substates [3, 6, 8].

Therefore, it is now interesting to compare these results to those obtained for the Na^+^ pump from *Indibacter alkaliphilus*, for which two O‐intermediates of similar absorption maxima close to 600 nm were already reported [61]. Fujisawa et al. studied these late intermediates in single‐beam RR experiments generating difference spectra from photostationary mixtures at different times after irradiation the sample for 1 s [36]. The authors reported two RR spectra, which they attributed to an early and late O‐state, denoted as O1 and O2, respectively. In fact, the O584 substates in the early O584(1) and late O584(2) spectra (Figure 8) reveal frequencies of the C=C stretching modes that agree very well with those of the reported O1 and O2 states [36]. Also, the kinetic data published for the Na^+^ pump of *I. alkaliphilus* [61] are compatible with the kinetics of the total O584 formation and decay determined in this work (Table 1). Accordingly, the decay constants for the reactions L504 → O584 of ca. 2 ms and O584 → KR525 of 5–7 ms refer to the formation of O1 and decay of O2, respectively (Table 1). The O2 state prevails in the TR RR spectrum at 12 ms, although its relative contribution has already dropped to less than 10% of the maximum intermediate concentration (Figure 7). Hence, the time constants of O2 formation and decay must be similar (5–7 ms).

According to the proposal by Unno's group for the Na^+^ pump of *I. alkaliphilus* [36], the O1 state still adopts a 13‐*cis* configuration which is then isomerized to a (distorted) all‐*trans* configuration in O2. The present RR spectroscopic results on KR2 are in line with this proposal. Furthermore, they are also compatible with crystallographic data on the O584 state which suggested a distorted all‐*trans* configuration of the retinal [21]. Evidently, in that study the late O2 state was captured and crystallized.

### 3.6 Hydrogen Bonding and Electrostatic Interactions of the Schiff Base in KR525

The Schiff base plays a key role in the ion pump mechanism of retinal proteins. For the sodium pump, it has been proposed that deprotonation of the Schiff base upon formation and reprotonation with the decay of L504/M449 ensure unidirectional Na^+^ transport [18, 38]. Thus, spectroscopic studies have paid special attention to elucidate structure and interactions of the Schiff base.

The most detailed RR spectroscopic analysis has been reported by Nakamura et al. [38], who studied KR2 in H_2_O, D_2_O, and a 1:1 mixture of H_2_O and D_2_O. The authors reported a small frequency shift of the C=NH^+^ stretching mode in the mixed buffer compared to H_2_O. These findings were taken as an evidence for a strong vibrational coupling of the C=NH^+^ stretching with the water bending mode. However, no effect on the bandshape of the C=NH^+^/C=ND^+^ was noted upon exchanging the buffer from H_2_O to D_2_O, such that vibrational energy transfer to a nearby water was ruled out [33, 38].

At first glance, these results and interpretations seem to be in contradiction with our findings. First, we do observe a significant reduction of the band width of the Schiff base stretching mode upon H/D exchange, which indicates a water molecule in close proximity to the Schiff base (Figure 4). Furthermore, the magnitude of the band narrowing is similar to that in BR570, which is consistent with the very similar frequencies, and corresponds to similar energy gaps between donor and acceptor (Section S3). Second, we have provided evidence for the existence of two conformational substates, both of them in the all‐*trans*, 15‐*anti* configuration. Both substates exhibit a distorted C(15)=N bond as indicated by the relative intensity and frequency of the C(14) HOOP mode. The only notable differences between the spectra with 458, 488, and 568 nm excitation are the position of the main C=C stretching mode and the band broadening of the C=NH^+^ stretching, concomitant to a small frequency shift. Thus, the difference between the two substates is ascribed to the electrostatic interactions of the Schiff base, which, in turn, affect the electronic transition. In view of the inverse correlation between the C=C stretching frequency and the wavelength of the absorption maximum [60], the low frequency component corresponds to a state with a red‐shifted absorption (substate I) compared to the high‐frequency component (substate II). Thus, the modes of substate II gain resonance enhancement upon excitation at the high energy side of the absorption band. This is reflected by the intensity ratio of the high‐frequency C=C stretching component at ca. 1540−1547 cm^−^^1^ with respect to the low‐frequency component at 1532 cm^−^^1^ that increases from 0.08 (568 nm) via 0.16 (488 nm) to 0.81 (458 nm).

Interestingly, in the RR studies of KR525 by the Kandori group [33, 38], 532 nm excitation was employed such that preferentially substate I was probed. However, only in substate II the C=N stretching shows a substantial band broadening in H_2_O (Figure 4), which therefore was not detected in the work of Kandori's group [33, 38]. Moreover, the exclusive use of a single excitation line in their studies impaired the detection of the second substate.

**FIGURE 9:**
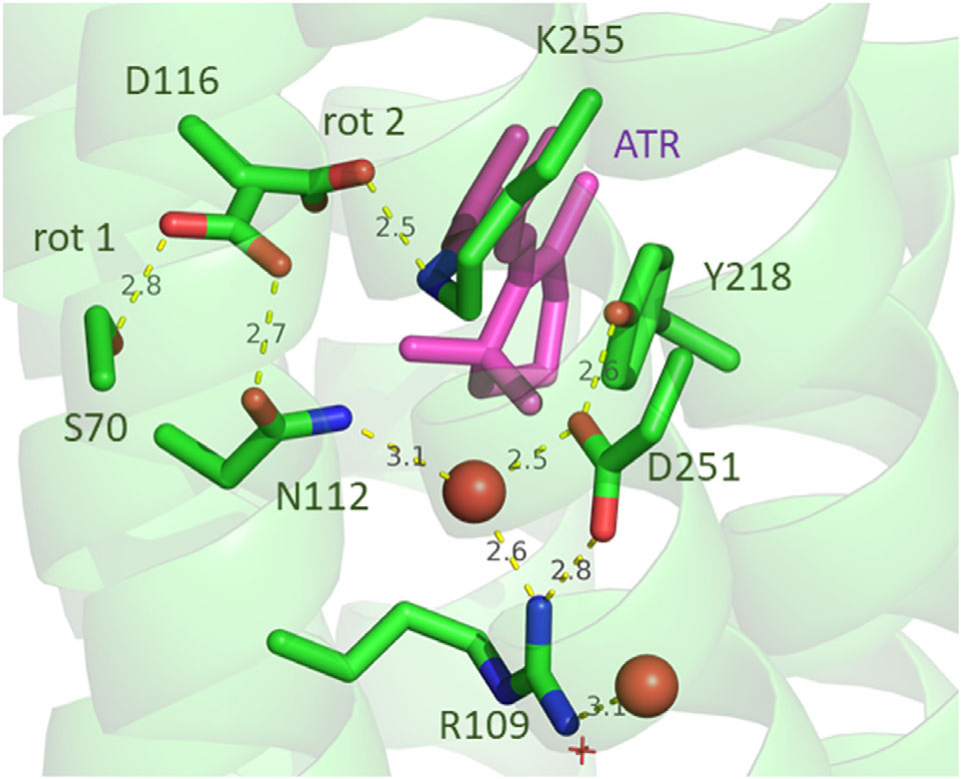
Close‐up view of the of Schiff base region of KR2 (PDB: 3X3C) [18] at neutral pH, showing two rotamers of D116 (rot I, rot II) and important amino acid residues as well as a structural water (red full circle). The retinal Schiff base chromophore is displayed in magenta. Hydrogen bonding distances are indicated. The presentation is similar to Figure 4b in ref. [18].

Next, we ask for the structural basis of the different electrostatic interactions and resultant shifts of the electronic transition in the two substates. Here, we refer to the crystal structure data presented by Kato et al., who demonstrated two different arrangements of the Schiff base and its counterion Asp116 [18]. The structure of monomeric KR2, obtained from neutral solutions and thus close to the conditions of the present and the reported RR experiments [38], displays two Asp116 rotamers (Figure 9). In one case, the carboxylate side chain points to the Schiff base (rotamer II) whereas the other rotamer interacts with Ser70 and Asn112 (rotamer I), thus impairing any direct or water‐mediated contact with the Schiff base. These results are consistent with the crystal structures of pentameric KR2 at different pH, indicating a transition from rotamer II to I upon decreasing the pH [20]. Kato et al. further demonstrated that at low pH (4.0), i.e., upon putative protonation of Asp116, the absorption spectrum is redshifted to 536 nm compared to 526 nm at pH 7.0 [18]. The acid form was then attributed to rotamer I, consistent with the acid‐induced redshift upon protonation of the counterion in BR [62]. Hence, we assign rotamer I to substate I and rotamer II to substate II.

In the region of the Schiff base, only one water molecule was detected, which is hydrogen bonded to Asn112, Asp251, and Arg109 (Figure 9) [18, 20, 23], but too far away from the Schiff base to account for vibrational energy transfer. We therefore assume that there is at least one more mobile water molecule in the vicinity of Asp116 that is shifted close to the Schiff base only in rotamer II such that the different vibrational energy transfer properties in the substates II and I can be understood. Interestingly, rotamer I and rotamer II are observed in KR2 pentamers and monomers, respectively, and pentamers seem to prevail in detergent‐solubilized KR2 [20, 23, 38, 63], to which the present and reported RR [38] measurements refer. However, it is very likely that, due to the low energy barriers, rotamer I and II represent limiting cases of a distribution of rotamers formed upon rotation around the Cα‐Cβ bond. Hence, the two substates may be taken as representatives of two populations rather than well‐defined and rigid conformations. This interpretation is supported by the frequency variation of the high‐frequency C=C stretching component with the excitation wavelength and the asymmetric bandshape of the C=N stretching at 458 nm.

### 3.7 Functional Implications

Membrane proteins that function as light‐driven ion pumps require switches that gate the uptake and the release of the ions [1]. These switches may be based on affinity or accessibility changes as discussed in detail for proton and anion pumps. For Na^+^ pumps such as KR2, two conformational gating steps have been proposed, which take place with the transition of L504/M449 to O584 (Na^+^ uptake) and the decay of O584 to the parent state KR525 (Na^+^ release) [4, 7, 23]. The first gating mechanism is associated with deprotonation of the Schiff base, which weakens the electrostatic interactions and possibly widens the space for accommodation of a dehydrated Na^+^ [23]. Based on the present results, we propose that this proton transfer from the Schiff base to Asp116 occurs in the substate II with the Asp116 side chain pointing toward the Schiff base. Furthermore, in substate II (but not in substate I) of KR525, one (or more) flexible water molecule(s) must be located in the vicinity of the Schiff base. Such water molecule(s) may help stabilize the bound Na^+^.

The release of Na^+^ is coupled to the 13‐*cis* → all‐*trans* reisomerization of the chromophore, which we have shown to occur between 5 and 7 ms (vide supra). In view of similar RR spectroscopic results and kinetics for *I. alkaliphilus* [35, 36, 64], we conclude that a conformational release switch is a general mechanistic feature of Na^+^ pumps. This implies that the early O584 substate, O1, formed upon re‐protonation of the Schiff base, adopts a 13‐*cis*, 15‐*anti* retinal configuration. Due to close proximity of the C=NH^+^ to the bound Na^+^, O1 is characterized by a high local electrostatic field, which was suggested to be the driving force the release of Na^+^ [7]. This final step of the pump mechanism, however, requires the retinal isomerization to the all‐*trans* configuration in O2. Hence, in both O2 and KR525, the chromophore adopts a protonated all‐*trans*, 15‐*anti* structure. Therefore, the main difference between these two states is the bound Na^+^ and the enhanced electrostatic interactions in O2, which may account for the lower C=C stretching mode at 1526 cm^−^^1^ compared to KR525 (1532 cm^−^^1^). Thus, the decay of O2 to KR525 mainly requires the release of Na^+^ and presumably only minor structural adjustments of the surrounding protein atoms.

## 4 Conclusions

We have employed pump‐probe TR RR spectroscopy on KR2 to probe the dark state and the temporal evolution of intermediates in the micro‐ and millisecond time range. Compared to single‐beam and photostationary techniques [15, 33], the present approach allows defining experimental parameters to ensure fresh‐sample condition and low photoconversion by the probe beam as well as a precise control of the delay time between pump and probe event. In this way, we could characterize two conformational switches that control the mechanism of Na^+^ pumping.

Na^+^ uptake in the M449 intermediate seems to take place within a specific arrangement of the Schiff base—Asp116 counterion—water complex that in the dark state was identified as substate II [18]. It is in equilibrium with substate I in which the side chain of Asp116 points away from the Schiff base. Given that this equilibrium persists also in M449, it is shifted toward substate II upon loading with Na^+^.

Na^+^ release occurs with the decay of O584 which was found to be a superposition of two intermediates formed within ca. 2 ms (O1) and 5–7 ms (O2), in analogy to *I. alkaliphilus* [36]. The transition from O1 to O2, which corresponds to the retinal isomerization from 13‐*cis* to all‐*trans*, opens the gate for the Na^+^ release, which, in turn, is presumably driven by electrostatic forces [7].

## Supporting Information

Additional supporting information can be found online in the Supporting Information section. **Supporting** **Figure S1.** Purification of KR2. A, SDS‐PAGE shows an overexpression at 25 kDa. B, UV vis absorption spectrum of the purified KR2 dark state at pH 8.0. The absorption ratio protein/chromophore was typically 2.0. **Supporting Figure S2.** Uncorrected RR spectrum of the dark state of KR2 incorporated in phospholipid vesicles, measured with 568 nm excitation (0.05 mW). The negative spikes in the grey‐shaded region (black arrow) were removed by in the spectrum shown in Figure 3 of the paper. **Supporting Figure S3.** RR spectra of KR525, obtained with 488 and 458 nm, in the C=C stretching region. The second derivatives do not allow identification of the positions band components. **Supporting Figure S4.** TR RR spectra of KR2 with 488 nm excitation of the parent state (probe only – top) and with the additional pump beam (568 nm) at different delay times. The parent state spectrum was analysed by a band fitting using Lorentzian lineshapes (dotted curves) which were then used to generate the component spectrum of KR525 (blue solid line). The TR RR spectra, including the component spectra of KR525 and L505 (red solid lines) are shown for two delay times. The grey traces represent the residuals of the overall fits. **Supporting Figure S5.** TR RR spectra of KR2 with 568 nm excitation of the parent state (probe only – top) and with the additional pump beam (514 nm) at different delay times. The parent state spectrum was analysed by a band fitting using Lorentzian lineshapes (dotted curves) which were then used to generate the component spectrum of KR525 (blue solid line). The TR RR spectra, including the component spectra of KR525 and O584 (magenta solid lines) are shown for two delay times. The grey traces represent the residuals of the overall fits. **Supporting Figure S6.** TR RR spectra of L504 obtained with 488 nm excitation as in Fig. S3. The spectra were obtained from the measured TR RR spectra at different delay times after subtracting the component spectrum of KR525. **Supporting Figure S7.** TR RR spectra of O584 obtained with 568 nm excitation as in Fig. S4. The spectra were obtained from the measured TR RR spectra at different delay times after subtracting the component spectra of KR525. The spectra O584(1) and O584(2) refer to TR RR measurements with delay times of 4.4 and 12 ms, respectively. The spectrum on top represents the ‘average’ intermediate spectrum summed over all delay times, essentially the sum of O584(1) and O584(2). **Supporting Table S1.** Spectral parameters of the Schiff base stretching in the dark states of retinal proteins.

## Funding

This work was supported by the Deutsche Forschungsgemeinschaft (221545957, 390540038), Einstein Stiftung Berlin (EVF 2018‐427).

## Conflicts of Interest

The authors declare no conflicts of interest.

## Supporting information

Supplementary Material

## Data Availability

The data that support the findings of this study are available from the corresponding author.
