# Supplementary material for: The Conformational Switches of a Bacterial Light‐Driven Sodium Pump Characterized by Time‐Resolved Resonance Raman Spectroscopy
Source: Chemphyschem. 2026 Apr 9;27(7):e202500905. doi: 10.1002/cphc.202500905 (PMC13066537; doi:10.1002/cphc.202500905)
Supplement: Supplementary file 1 — Supplementary Material [file CPHC-27-e202500905-s001.pdf]

Supporting Information

The conformational switches of a bacterial light-driven sodium pump characterized by time-resolved resonance Raman spectroscopy

Anna Lena Schäfer,[1] Arita Silapetere, [2] Peter Hegemann,[2] Katrina T. Forest,[3] Uwe Kuhlmann,[1] Peter Hildebrandt[1*]

[1] Technische Universität Berlin, Institut für Chemie, Straße des 17. Juni 135, D-10623 Berlin, Germany

[2] Humboldt-Universität zu Berlin, Institut für Biologie, Invalidenstraße 42, D-10115 Berlin, Germany

[3] University of Wisconsin-Madison, Department of Bacteriology, 1550 Linden Dr., Madison, WI 53706, USA

1. Materials

1.1. Protein expression and purification

Overexpression and purification follows previously published procedures with minor modifications.[1,2] The expression plasmid (pet21a+) was transformed into C41(DE3) *E. coli* cells. Cells were stored on LB-Agar plates with appropriate selection antibiotic (Ampicillin - 100 µg/ml). A small overnight liquid pre-culture (20 –50 ml) stemming from one colony from the LB-Agar plate was cultivated in an Erlenmeyer flask with appropriate selection antibiotics (Ampicillin – 100 µg/ml) for 12–18 h shaking with 190 rpm at 37 °C. Subsequently, the large main culture was incubated under the same conditions until OD600 ≈ 0.6. Then, expression was induced by adding 0.5 mM isopropyl β-D-thiogalactopyranoside (IPTG) and the culture was supplemented with 5 µM all-trans-retinal. The cells were harvested by centrifugation (6000 x g, 10 min, 4 °C) 3 h after induction. The resulting cell pellet was resuspended in an appropriate amount of buffer (~10 ml/g cells) and frozen in liquid nitrogen. All following steps were performed under red-light. Cells were thawed and DNAse I was added. The cells were then broken by 5 passages through a French pressure cell. To remove cell debris, the cells were centrifuged (1500 x g, 10 min, 4°C) and the supernatant containing the membrane protein was transferred to new centrifuge tubes. It was again centrifuged (150000 x g, 1h, 4°C) and the supernatant was discarded. The pellet containing the KR2 was scraped from the tube and transferred to a dounce glass homogenizer for shearing cells including a few milliliters of disruption buffer (excl. detergent), where it was mixed for 5 min on ice. The solution was then transferred to a falcon tube, and DDM and CHS were added to a working concentration of 1.5 % DDM and 0.3 % CHS plus appropriate amount of disruption buffer. The tube was wrapped in aluminum foil to avoid interaction with light, and left shaking softly over night at 4 °C. The sample was filled into centrifugation tubes and centrifuged (150000 x g, 1.5 h, 4 °C). The supernatant was loaded onto a 5 mL Ni-NTA binding column (GE healthcare) previously equilibrated with the binding buffer. It was washed with 10 CV wash buffer, then eluted with

elution buffer. The eluted volume was then dialyzed for 2 h using a dialysis-tube with an appropriate molecular cut off value of (6500-8000 kDa) and a dialysis buffer volume around 300 x the sample volume. The purified KR2 sample was concentrated to 100 - 150 µM, frozen with liquid nitrogen, and stored at −86 °C. The purity and quality of the sample was monitored using SDS-PAGE, and UV-Vis absorption spectroscopy (Fig. S1).

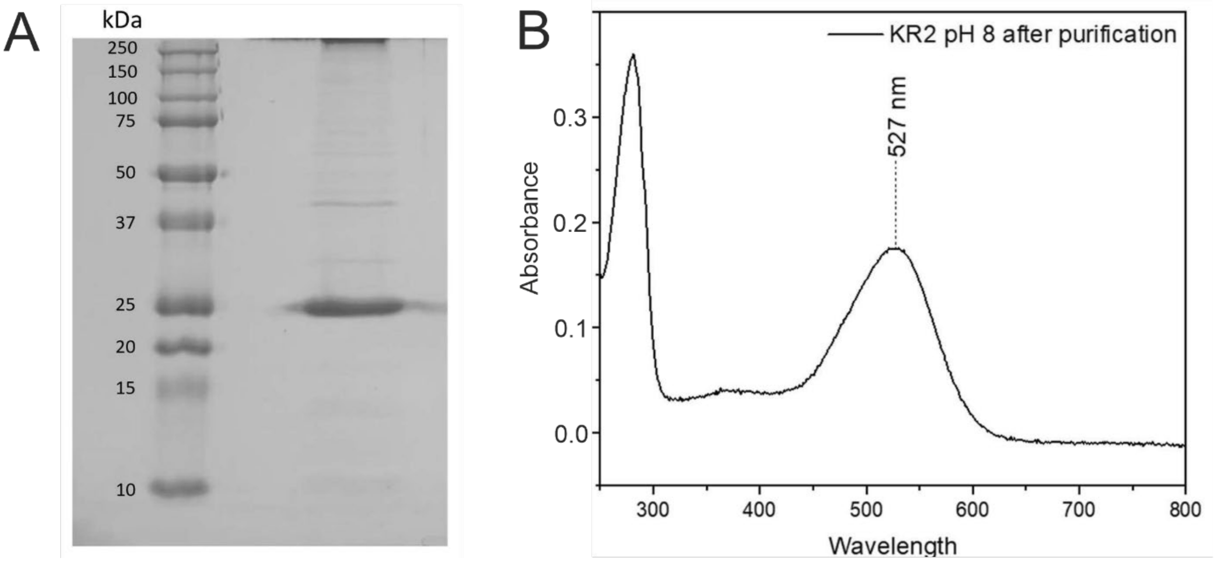

Figure S1. Purification of KR2. A, SDS-PAGE shows an overexpression at 25 kDa. B, UV vis absorption spectrum of the purified KR2 dark state at pH 8.0. The absorption ratio protein/chromophore was typically 2.0.

1.2. Liposomes

Unilamellar vesicles were prepared using POPC or POPG. The lipids were dissolved in $CHCl_3$ to a final concentration of 0.5 mg/mL in a volume of 100 µL and were subsequently dried using N_2, followed by vacuum dehydration overnight. The resulting lipid multilayers were covered with 500 µL of buffer to swell and resolve for 10 min, followed by three cycles of vortex for 60 sec with 10 min breaks in between each cycle to allow for further rehydration. The vesicle solution was then passed through an extruder with a polycarbonate filter (pore size 100 nm) for 31 times, resulting in unilamellar vesicles with a diameter of 100 nm. For KR2 incorporation,

the phospholipid vesicles were destabilized using detergent (0.5 mM DDM/CHS-mixture). From a stock solution of 1 mg/mL solubilized protein up to 10 % of the total volume was added to the destabilized vesicle solution, followed by incubation and gentle shaking for 4 h. The sample was then transferred to a dialysis tube (6500-8000 kDa cutoff) and placed into buffer with no detergent. Additionally, biobeads (BioRad) were added to the dialysis buffer. Dialysis was performed for several days (4 - 5 days, softly stirring at 4 °C), thereby slowly removing any detergent. Buffer and beads were exchanged daily. The sample was then removed from the dialysis tube.

2. Resonance Raman Spectroscopy

2.1. Experimental parameter for probing the dark state and intermediates

A detailed analysis of the experimental parameters for pump-probe TR RR experiments with a confocal spectrometer set-up is given elsewhere.[3]

2.1.1. Probe-only experiments

The residence time of the sample in the probe beam is given by

(S1) $$\Delta t = \frac{r_R}{\pi R \nu_0}$$

where r_R is the radius of the Raman probe beam. Here and in the following, the laser beam radius is defined by the drop of the laser intensity from its maximum value I_0 to I_0/e^2. Using a rotating cell of 13 mm radius and a rotational frequency of 20 s^{-1} which is sufficient to fulfill the fresh-sample condition ($\frac{k_{RL}}{\nu_0} \geq 4$), the residence time of the sample in the probe beam (r_R = 2 µm) is $\Delta t = 2.4 \cdot 10^{-6} s$.

The photochemical rate constant for the conversion of parent state to the primary photoproduct within an irradiated volume element passing the laser beam, $l_0(t)$, is given by

(S2) $$l_0 = 0.481 \cdot \gamma \cdot \varepsilon(\lambda) \cdot \lambda \cdot \frac{P}{r_R^2}$$

where γ, λ, and $\varepsilon(\lambda)$ denote the photochemical quantum yield, the wavelength of the Raman probe beam (in nm), and the extinction coefficient of the retinal chromophore at λ (in $L \cdot mol^{-1} \cdot cm^{-1}$), respectively. Here P is expressed in mW and r_R in µm and thus l_0 in s^{-1}.

The quantum yield γ for KR2 are not known such that we have chosen an average value from microbial rhodopsins for the forward KR525 →K563 and the reverse reaction of 0.3 and 0.85, respectively.[4–7] Furthermore, we use extinction coefficients for KR525 of 10000, 30000, and 15000 $L \cdot mol^{-1} \cdot cm^{-1}$ at 458, 488, and 568 nm excitation, respectively. The corresponding values for K563 were assumed to be 3300, 10000, and 50000 $L \cdot mol^{-1} \cdot cm^{-1}$ for 458, 488,

and 568 nm excitation, respectively. The photoproduct K563 may either thermally decay to the L intermediate with an approximate rate constant of k_K, or it converts back to BR photochemically (l_B), as depicted in Fig. 1 of the manuscript. The rate constant l_B is estimated in analogy to l_0 With a probe beam radius of 2 µm Eq. (S2) we evaluate the photochemical rate constants yields l_0 and l_B to

(S3a) $l_0(458\,nm) = 2.75 \cdot 10^5 \cdot P$ and $l_B(458\,nm) = 0.95 l_0(458\,nm)$

(S3b) $l_0(488\,nm) = 6.40 \cdot 10^3 \cdot P$ and $l_B(488\,nm) = 0.95 l_0(488\text{ nm})$

(S3c) $l_0(568\,nm) = 5.00 \cdot 10^3 \cdot P$ and $l_B(568nm) = 9.4 l_0(568\,nm)$

For 458 and 488 nm we have chosen $P = 0.1\,mW$. Thus we calculate a relative concentration for KR525 using the same value for the quantum yield (0.5) but an extinction coefficient Thus, we obtain $l_B = 0.33 l_0$ for 458 and 488 nm excitation but $l_B = 3.3 l_0$ for 568 nm excitation. With a rate constant of $3.7 \cdot 10^4 s^{-1}$ for the decay from K563 to L504 one can then calculate the concentration of the parent state KR525 at the end of the Raman probe event $[KR525]_f$ for different laser powers (see Supporting Information of ref. 3). Thus we obtained 0.96 and 0.89 for $[KR525]_f$ at 458 and 488 nm excitation, respectively. The relative concentration of KR525 that is measured during the residence time in the probe beam $[KR525]_{av}$ is then given by

(S4) $[KR525]_{av} = 0.5 \cdot (1 + [KR525]_f)$

and yields 0.98 and 0.95 for 458 and 488 nm, respectively. Due to the slow decay to L504, the remaining fraction of the protein is in the K563 state. Due to the red-shifted absorption, its contribution to the RR spectra can be neglected.

For 568 nm excitation, we have used 1 mW, corresponding to n$[KR525]_f = 0.84$ and thus to$[KR525]_{av} = 0.92$. In this case, the remaining fraction of 0.08 is in the K563 state. Here a small contribution of K563 in the RR spectrum cannot be ruled due to the good resonance conditions.

2.2. Pump-probe experiments

The probe beam parameters and rotational frequency of the cell were the same in single-beam (probe-only) and pumo-probe experiments. The pump-probe experiments were carried with 488 and 568 nm excitation, using 568 and 514.5 nm pump beams, respectively. In the both cases, the pump radius was adjusted to 100 μm with 100 mW and 500 mW for the 568 and 514.5 nm pump beams, respectively.

3. Resonance Raman spectra

3. 1. Resonance Raman spectrum of KR525 in liposomes

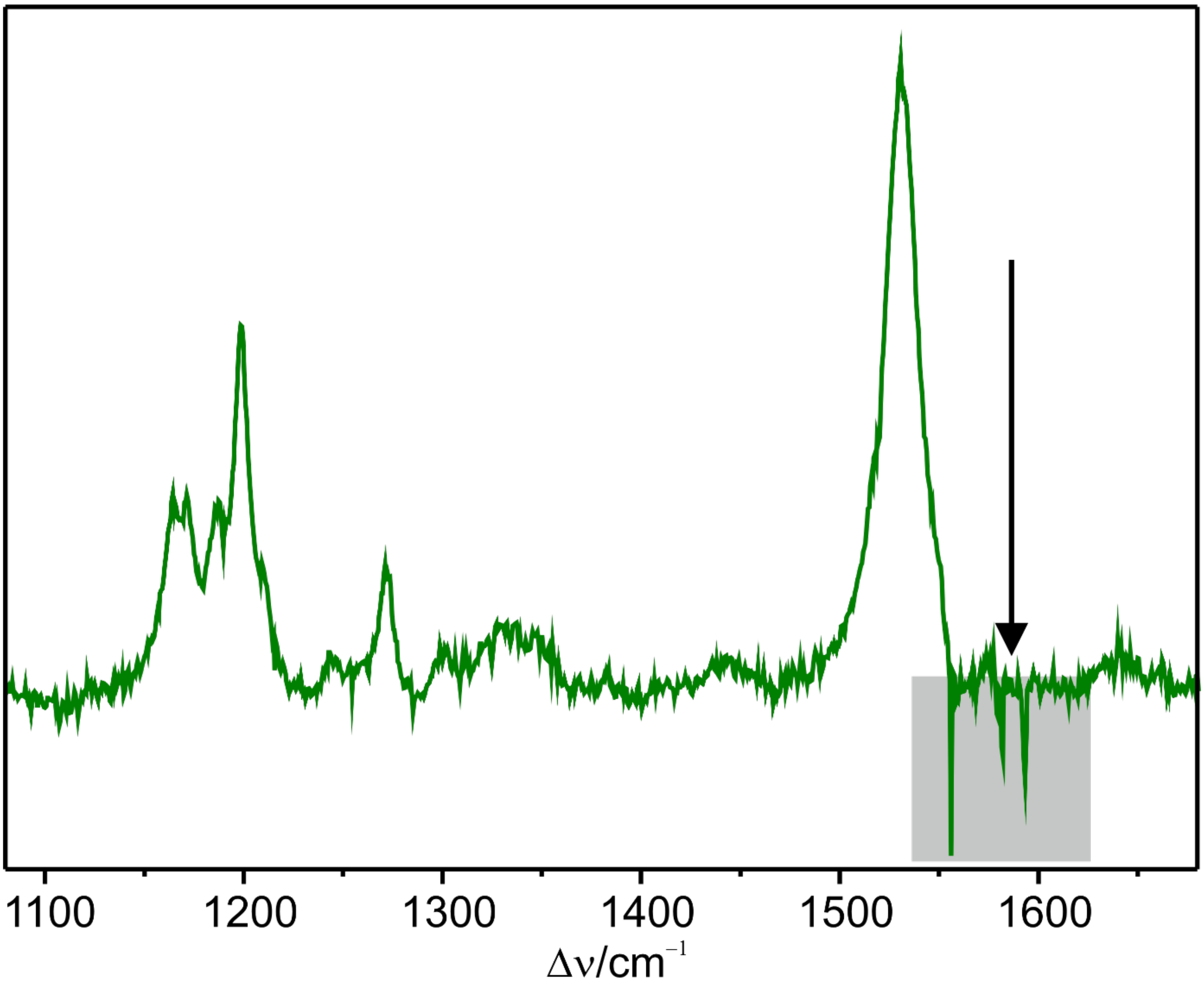

Figure S1. Uncorrected RR spectrum of the dark state of KR2 incorporated in phospholipid vesicles, measured with 568 nm excitation (0.05 mW). The negative spikes in the grey-shaded region (black arrow) were removed by in the spectrum shown in Figure 3 of the paper.

3.2. Probe only spectra

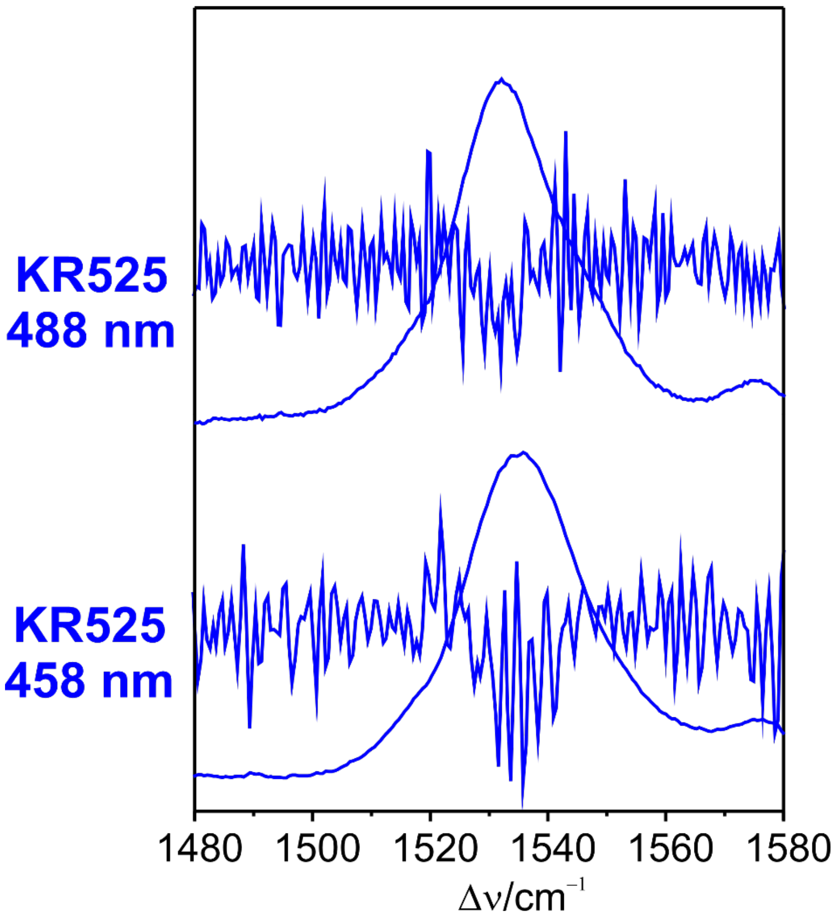

Figure S2. RR spectra of KR525, obtained with 488 and 458 nm, in the C=C stretching region. The second derivatives do not allow identification of the positions band components.

3.3. Pump-probe time-resolved resonance Raman spectra

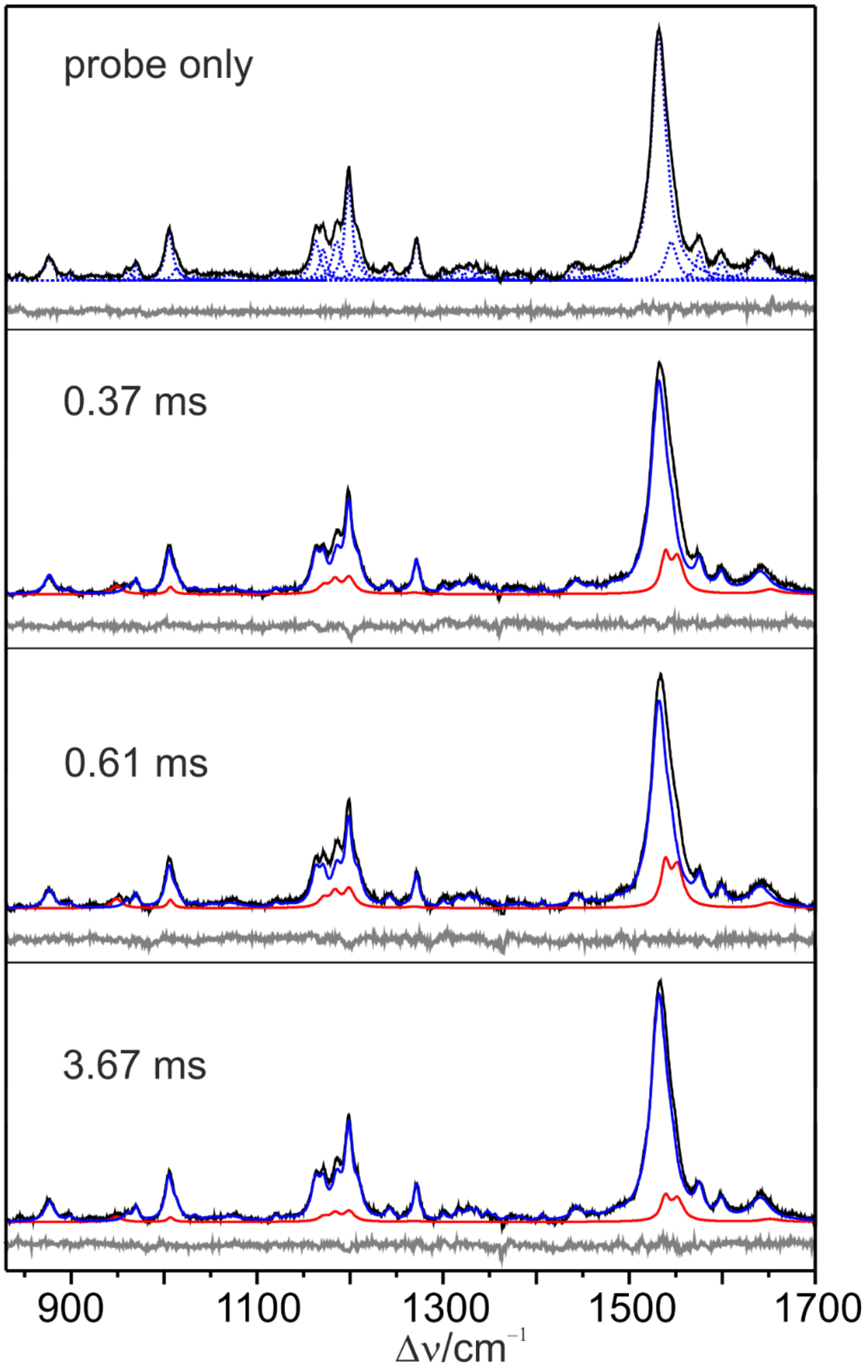

Figure S3. TR RR spectra of KR2 with 488 nm excitation of the parent state (probe only – top) and with the additional pump beam (568 nm) at different delay times. The parent state spectrum was analysed by a band fitting using Lorentzian lineshapes (dotted curves) which were then used to generate the component spectrum of KR525 (blue solid line). The TR RR spectra, including the component spectra of KR525 and L505 (red solid lines) are shown for two delay times. The grey traces represent the residuals of the overall fits.

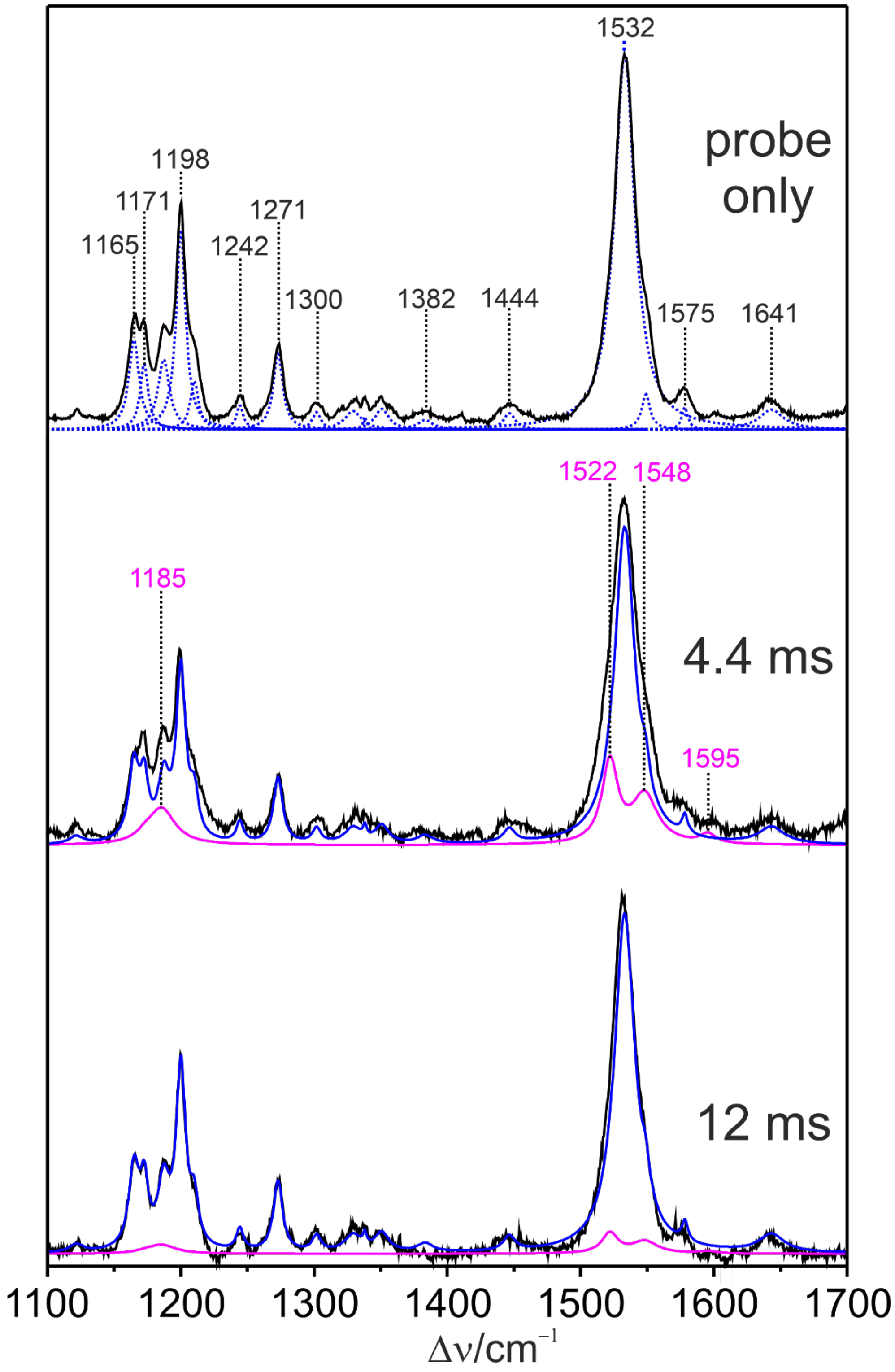

Figure S4. TR RR spectra of KR2 with 568 nm excitation of the parent state (probe only – top) and with the additional pump beam (514 nm) at different delay times. The parent state spectrum was analysed by a band fitting using Lorentzian lineshapes (dotted curves) which were then used to generate the component spectrum of KR525 (blue solid line). The TR RR spectra, including the component spectra of KR525 and O584 (magenta solid lines) are shown for two delay times. The grey traces represent the residuals of the overall fits.

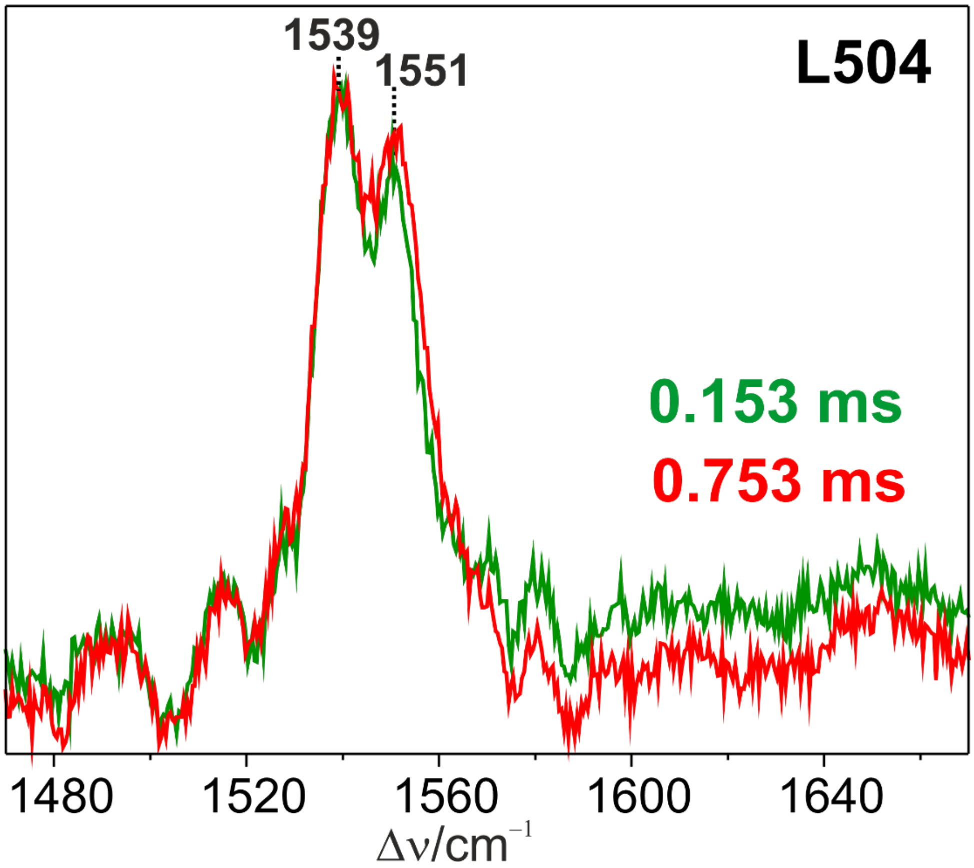

Figure S5. TR RR spectra of L504 obtained with 488 nm excitation as in Fig. S3. The spectra were obtained from the measured TR RR spectra at different delay times after subtracting the component spectrum of KR525.

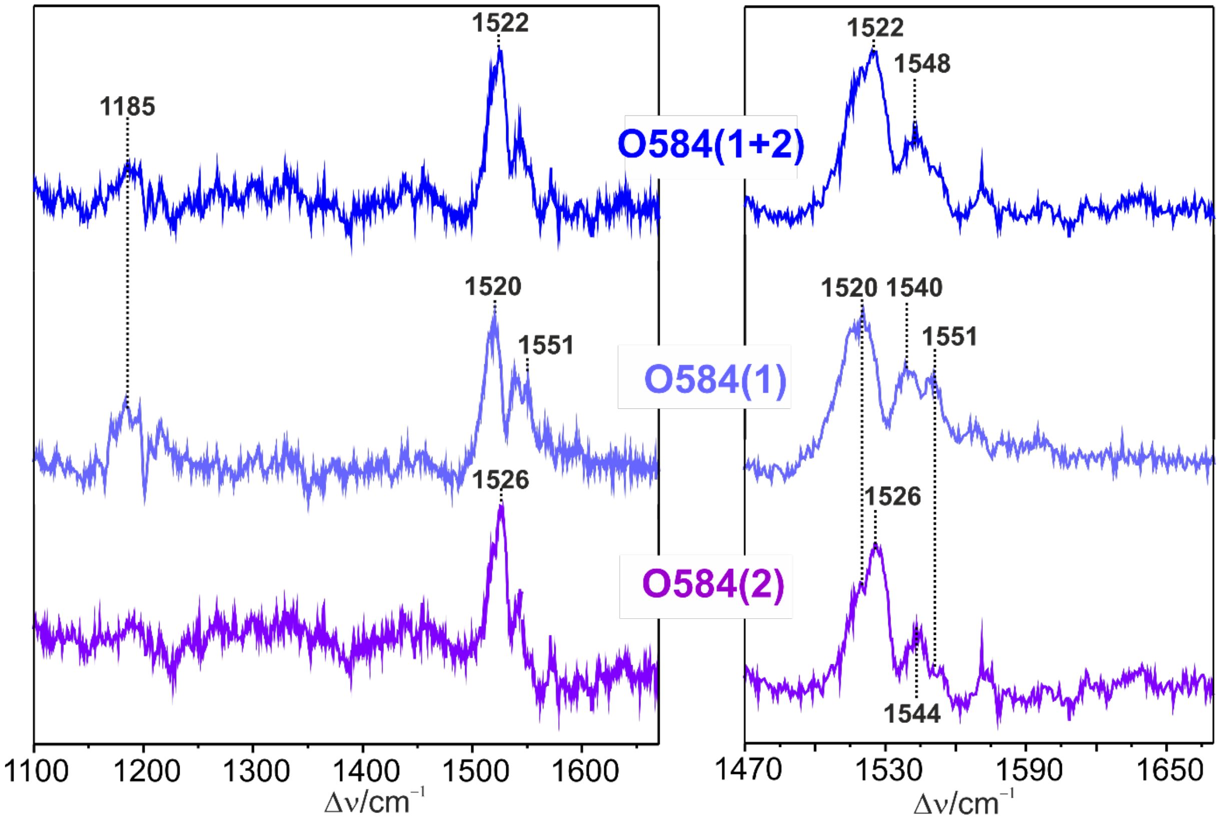

Figure S6. TR RR spectra of O584 obtained with 568 nm excitation as in Fig. S4. The spectra were obtained from the measured TR RR spectra at different delay times after subtracting the component spectra of KR525. The spectra O584(1) and O584(2) refer to TR RR measurements with delay times of 4.4 and 12 ms, respectively. The spectrum on top represents the "average" intermediate spectrum summed over all delay times, essentially the sum of O584(1) and O584(2).

3.4. Vibrational energy transfer

The lifetime Δt of a vibrational transition is given by

(S1) $$\Delta t = \frac{1}{4\pi \cdot \Delta \nu \cdot c}$$

where $\Delta \nu$ is the half width of the vibrational band and c the velocity of light. We assume that energy transfer occurs from the Schiff base to an adjacent water molecule. Then the rate constant for vibrational energy transfer k_{ET} is given by

(S2) $$k_{ET} = \frac{1}{\Delta t(H_2O)} - \frac{1}{\Delta t(D_2O)}$$

The data is listed in Table S1.

Table S1. Spectral parameters of the Schiff base stretching in the dark states of retinal proteins

Protein/state/configuration	Buffer	ν(C=N)/ cm^{-1}	Δν/cm^{-1}	$\Delta t/10^{-13}s$	$k_{ET}/10^{12}s^{-1}$
KR2 (this work)					
KR525, all-trans, 15-anti	H_2O	1639.8	27.8	0.95	1.85
	D_2O	1617.0	22.9	1.16	
ChR2[8]					
DA, all-trans, 15-anti	H_2O	1661.1	18.7	1.82	1.54
	D_2O	1631.8	14.6	1.42	
DA, 13-cis, 15-syn	H_2O	1630.8	32.6	0.81	3.94
	D_2O	1622.1	22.2	1.20	
BR[9]					
BR570, all-trans, 15-anti	H_2O	1641	16.3	1.63	1.41
	D_2O	1623	12.5	2.12	

References

[1] H. E. Kato, K. Inoue, R. Abe-Yoshizumi, Y. Kato, H. Ono, M. Konno, S. Hososhima, T. Ishizuka, M. R. Hoque, H. Kunitomo, J. Ito, S. Yoshizawa, K. Yamashita, M. Takemoto, T. Nishizawa, R. Taniguchi, K. Kogure, A. D. Maturana, Y. Iino, H. Yawo, R. Ishitani, H. Kandori, O. Nureki, *Nature* **2015**, *521*, 48–53.

[2] C. Grimm, A. Silapetere, A. Vogt, Y. A. Bernal Sierra, P. Hegemann, *Sci. Rep.* **2018**, *8*, 1–12.

[3] A. L. Schäfer, C. Gellini, R. Diller, K. T. Forest, U. Kuhlmann, P. Hildebrandt, **2025**, *submitted*.

[4] O. P. Ernst, D. T. Lodowski, M. Elstner, P. Hegemann, L. S. Brown, H. Kandori, *Chem. Rev.* **2014**, *114*, 126–163.

[5] A. Cheminal, J. Léonard, S. Y. Kim, K. H. Jung, H. Kandori, S. Haacke, *Phys. Chem. Chem. Phys.* **2015**, *17*, 25429–25439.

[6] R. Misra, I. Das, A. Dér, G. Steinbach, J. G. Shim, W. Busse, K. H. Jung, L. Zimányi, M. Sheves, *Chem. Sci.* **2023**, *14*, 9951–9958.

[7] M. A. Ostrovsky, O. A. Smitienko, A. V. Bochenkova, T. B. Feldman, *Biochem.* **2023**, *88*, 1528–1543.

[8] S. Bruun, D. Stoeppler, A. Keidel, U. Kuhlmann, M. Luck, A. Diehl, M. A. Geiger, D. Woodmansee, D. Trauner, P. Hegemann, H. Oschkinat, P. Hildebrandt, K. Stehfest, *Biochemistry* **2015**, *54*, 5389–5400.

[9] P. Hildebrandt, M. Stockburger, *Biochemistry* **1984**, *23*, 5539–5548.
